# A Novel Chaos-inspired Artificial Intelligence-based Model for Keratoconus Prediction

**DOI:** 10.18502/jovr.v21.18172

**Published:** 2026-07-31

**Authors:** Soheil Adib-Moghaddam, Nader Nassiri, Moein Bahman, Majid Mohebbi, Kourosh Sheibani

**Affiliations:** ^1^Universal Council of Ophthalmology (UCO), Tehran, Iran; ^2^Imam Hossein Medical Center, Shahid Beheshti University of Medical Sciences, Tehran, Iran; ^3^Bina Eye Hospital, Tehran, Iran; ^4^Basir Eye Health Research Center, Iran University of Medical Sciences, Tehran, Iran

**Keywords:** Artificial Intelligence, Chaotic Modeling, Keratoconus, Prediction

## Abstract

**Purpose:**

To develop a novel chaos-inspired artificial intelligence (AI)-based system for keratoconus prediction.

**Methods:**

We constructed, trained, and tested our prediction model using corneal tomography data obtained from Pentacam and Sirius imaging systems. The study included 65 healthy individuals and 48 patients with keratoconus recruited from a private ophthalmology clinic and Bina Eye Hospital in Tehran, Iran. Model development included stratified partitioning of the data into training and testing sets, followed by performance evaluation using standard classification metrics. Scheimpflug-based tomographic parameters were preprocessed, normalized, and analyzed using a chaos-inspired tensor decomposition framework.

**Results:**

Using Pentacam data, the model achieved sensitivity and specificity values of 88% and 90%, respectively. When using Sirius data, the sensitivity and specificity increased to 92% and 96%, respectively. Performance metrics were reproducible across repeated random train–test splits, indicating model stability. The chaos-inspired AI-based model demonstrated balanced sensitivity and specificity across both imaging systems, supporting robust discrimination between keratoconus and normal eyes.

**Conclusion:**

Given the complex and nonlinear behavior of keratoconus progression, influenced by biomechanical, genetic, and environmental interactions, we developed a novel chaos-inspired AI-based system (Iran Model) capable of accurately detecting keratoconus. Although the findings are promising, further validation in larger cohorts is required.

##  INTRODUCTION

Keratoconus (KCN) is an irreversible, asymmetric, bilateral corneal degeneration characterized by progressive stromal thinning and corneal protrusion, resulting in a cone-shaped cornea and irregular astigmatism.^[1]^ It is the leading global indication for corneal transplantation.^[2]^ The reported prevalence in the United States is 0.17 per 1000 individuals, but incidence appears to be rising across age groups and races, partly due to improved imaging and greater awareness.^[2]^ Despite decades of study, the etiology and risk factors of KCN are not fully understood.^[3]^

KCN presents with a broad spectrum of clinical manifestations, ranging from clinically evident disease to forme fruste KCN, a subclinical and incomplete manifestation characterized by subtle topographic and tomographic abnormalities without overt clinical signs. Progressive thinning and protrusion cause irregular astigmatism and myopia that impair vision.^[4]^ Patients in subclinical stages may retain normal acuity with few slit-lamp signs, leading to delayed diagnosis.^[5]^ Because KCN is progressive, early detection is vital to disease management. Depending on severity, treatment may range from conservative measures, rigid contact lenses, or refractive correction to intracorneal ring segments, toric intraocular lenses, or corneal collagen cross- linking.^[6,7]^ Early recognition also aids in assessing risk for post-refractive surgical complications. Since the onset often occurs in adolescence and worsens thereafter, it is imperative to have reliable tools for early diagnosis.^[4,8]^

Corneal imaging is the cornerstone of early KCN diagnosis.^[9]^ While traditional Placido disc topography and anterior OCT are useful, Scheimpflug-based tomography provides three-dimensional characterization of anterior and posterior surfaces, as well as pachymetry.^[3,10]^ Instruments such as Pentacam (Oculus, Optikgeräte GmbH, Wetzlar, Germany) and Sirius (CSO, Florence, Italy) yield reproducible topographic and pachymetric indices.^[9,11]^ These technologies underpin indices such as the Belin/Ambrósio deviation (BAD-D) and the Pentacam random forest index (PRFI), which improve sensitivity in distinguishing subclinical KCN from normal corneas, though diagnostic uncertainty persists in borderline cases.

Parallel advances in artificial intelligence (AI) have expanded ophthalmic diagnostic capabilities. Machine learning (ML) has been applied successfully to diabetic retinopathy, ${}^{\left[12-14\right]}$ retinopathy of prematurity,^[15]^ age-related macular degeneration,^[16]^ uveitis,^[17]^ cataract surgery,^[18]^ and glaucoma.^[19,20]^. In KCN, ML has been used to separate overt KCN, suspects, and normal eyes using tomographic maps.^[21]^ These models offer promise but face challenges such as overfitting to specific datasets, limited generalizability, and “black-box” decision-making.^[21]^

Chaos theory may provide a new framework for addressing these challenges. It describes deterministic systems that exhibit extreme sensitivity to initial conditions, yielding complex yet structured dynamics, the so-called “butterfly effect.”^[22,23]^ Such behavior is found in weather, fluid dynamics, and biological systems.^[24]^ Ocular physiology likewise demonstrates chaotic features. Hampson et al^[25]^ reported low-dimensional strange attractors in ocular aberration dynamics, indicating deterministic yet complex visual system behavior. In another study, accommodative mechanisms in myopic eyes showed chaotic fluctuations, and chaos-based metrics outperformed conventional measures of stress detection.^[26]^ Fan et al^[27]^ modeled irregular optical cavities and confirmed chaotic light dynamics and, thus, provided an analogy for how microstructural corneal changes might amplify biomechanical instability in KCN.

These findings support the biological plausibility of chaos-inspired modeling for the eye. Incorporating chaos theory into an AI framework may capture unstable and interdependent corneal behaviors more effectively than linear approaches, especially for early disease detection. Following the hypothesis that ocular biomechanics exhibit nonlinear and chaotic behavior, this study developed and evaluated a novel chaos-inspired machine-learning model for KCN detection, aiming to improve diagnostic accuracy and reduce limitations of existing approaches, such as overfitting.

##  METHODS

This study presents a novel AI-based model for KCN detection using tensor decomposition and chaos-inspired representation. The concept and methodology of the chaotic AI-based system for KCN prediction presented in this study (Iran Model) were conceived and developed by the first author (SAM). The methodology involved five stages: data acquisition, tensor construction, decomposition using Parallel Factor Analysis 2 (PARAFAC2), feature extraction, and classification.

The study was conducted at a private ophthalmology and optometry clinic and Bina Eye Hospital in Tehran, Iran, from 2020 to 2022. All participants provided written informed consent before entering the study. We also adhered to the Declaration of Helsinki when conducting this study.

A total of 226 eyes of 113 participants were included in this study. Of these participants, 62 were male, and 51 were female, with ages ranging from 23 to 74 years. The participants were categorized into two main subgroups: the first group included 65 healthy individuals (controls), while the second group consisted of 48 patients diagnosed with KCN (cases).

The study population comprised individuals with KCN and healthy participants. The KCN group included patients with varying disease severity, ranging from mild to severe. A comprehensive medical and ophthalmic history was obtained for each participant, followed by slit-lamp biomicroscopy and ophthalmoscopic examination.

Scheimpflug camera-based imaging was performed using Pentacam (OCULUS Optikgeräte GmbH) and Sirius (Costruzione Strumenti Oftalmici) devices. Participants were classified as either KCN or normal based on clinical examination and ABCD criteria.^[28]^ The ABCD criteria diagnose KCN using thickness, anterior/posterior curvature, biomechanics, clinical signs, and disease progression, which provide a comprehensive assessment for early detection and monitoring.^[28]^ All participants were also screened for dry eye syndrome and, if needed, received treatment at least 4 weeks prior to imaging.

The dataset was derived from clinical records. It comprised imaging data containing 34 samples and 58 features. Missing values were addressed through mean imputation based on the available incomplete records. Outliers were detected and managed using z-score thresholding.The data underwent a series of preprocessing steps to enhance its quality and ensure compatibility with the subsequent analysis pipeline. These procedures were crucial for minimizing bias, improving model performance, and maintaining consistency across the dataset. First, all numerical features were scaled to a range between 0 and 1 using min–max normalization. This step ensured that variables with differing units and magnitudes were adjusted to a common scale, preventing any single feature from disproportionately influencing the model. Next, categorical variables were transformed through label encoding, converting text-based categories into numerical values suitable for processing by ML algorithms. To further optimize the dataset, we performed correlation analysis for feature selection. Highly correlated features were carefully reviewed, and those contributing minimally to the target variable were excluded to reduce dimensionality, enhance computational efficiency, and improve interpretability. Lastly, data cleaning was performed by identifying and removing duplicate entries to ensure data integrity and prevent potential overrepresentation or leakage in the modeling process.The exclusion criteria included individuals with ocular conditions other than KCN, such as corneal degenerations, connective tissue diseases, strabismus, cataract, and retinal and macular diseases, as well as any prior refractive surgery.

### Diagnostic Criteria and Device Role

All patients underwent comprehensive clinical evaluation, including slit-lamp biomicroscopy, refraction, and ophthalmoscopy. The diagnosis of KCN was established according to the ABCD grading system proposed by Belin et al,^[28]^ which integrates anterior and posterior curvature, thinnest pachymetry, and corrected distance visual acuity. Pentacam tomography served as the primary platform to classify participants into the KCN and healthy groups, while Sirius tomography was employed as a confirmatory device to strengthen diagnostic robustness. In cases of discrepancy between Pentacam and Sirius outputs, the final diagnosis was determined by the consensus of two experienced corneal specialists, with priority given to clinical findings and Pentacam ABCD staging as recommended in the literature. No patient was assigned to the KCN group solely on the basis of automated machine classification without supportive clinical evidence.

#### Machine learning (ML) model development

We developed two ML models, one using Sirius data and the other using Pentacam data, to identify nonlinear relationships among corneal imaging parameters.

Furthermore, inspired by the capabilities of the PARAFAC 2 model to track moving sources over time, we applied the PARAFAC2 decomposition to capture latent patterns in corneal parameters while allowing individual variation across patients.

The PARAFAC 2 model is a generalization of the standard PARAFAC model designed to handle multi-way data with one mode that varies in size across samples, making it particularly useful for analyzing irregular or longitudinal data.^[26]^ Unlike PARAFAC, which assumes fixed factor matrices across all dimensions, PARAFAC2 allows one mode (e.g., time or subjects) to change while maintaining constraints that preserve interpretability.^[29]^ This flexibility makes it ideal for applications in fields like neuroscience, healthcare, and chemometrics, where data may have varying lengths or shifting patterns.^[30]^ Despite this adaptability, PARAFAC2 still provides a unique and meaningful decomposition, enabling insightful multi-way data analysis.^[31]^ In the context of KCN, this flexibility allows us to model inter-subject variation in corneal structure while preserving shared dynamics across corneal parameters. These are known to evolve in a nonlinear and patient-specific manner.

To implement this framework, we followed a systematic series of steps to diagnose KCN and examine dynamic relationships. Each patient's corneal parameters were arranged into a matrix, where rows represented individual features and columns represented structured groupings of imaging parameters that reflected internal variation in corneal measurements. These patient-specific matrices were stacked into a third-order tensor, capturing variability across features, observations, and patients. This allowed us to analyze dynamic interactions among corneal parameters and generate individualized parameter interaction maps.

To prepare the dataset for model training and evaluation, it was divided into two distinct subsets: 60% for training and 40% for testing. This partitioning was performed using stratified random sampling to preserve the original distribution of KCN and control participants across both subsets, thereby minimizing potential class imbalance that could bias the model. To ensure the integrity of the evaluation process, the training and testing sets were made completely independent, with no overlapping samples. This separation guaranteed that the performance metrics would reflect the model's generalizability to unseen data rather than its ability to memorize specific cases.

To further strengthen model training and minimize overfitting, k-fold cross-validation was performed within the training dataset. In this process, the training data were divided into k equal subsets. The model was iteratively trained on k - 1 folds and validated on the remaining fold, ensuring that each subset served once as the validation dataset. The configuration demonstrating optimal validation performance was subsequently evaluated using the independent test set.

**Figure 1:**
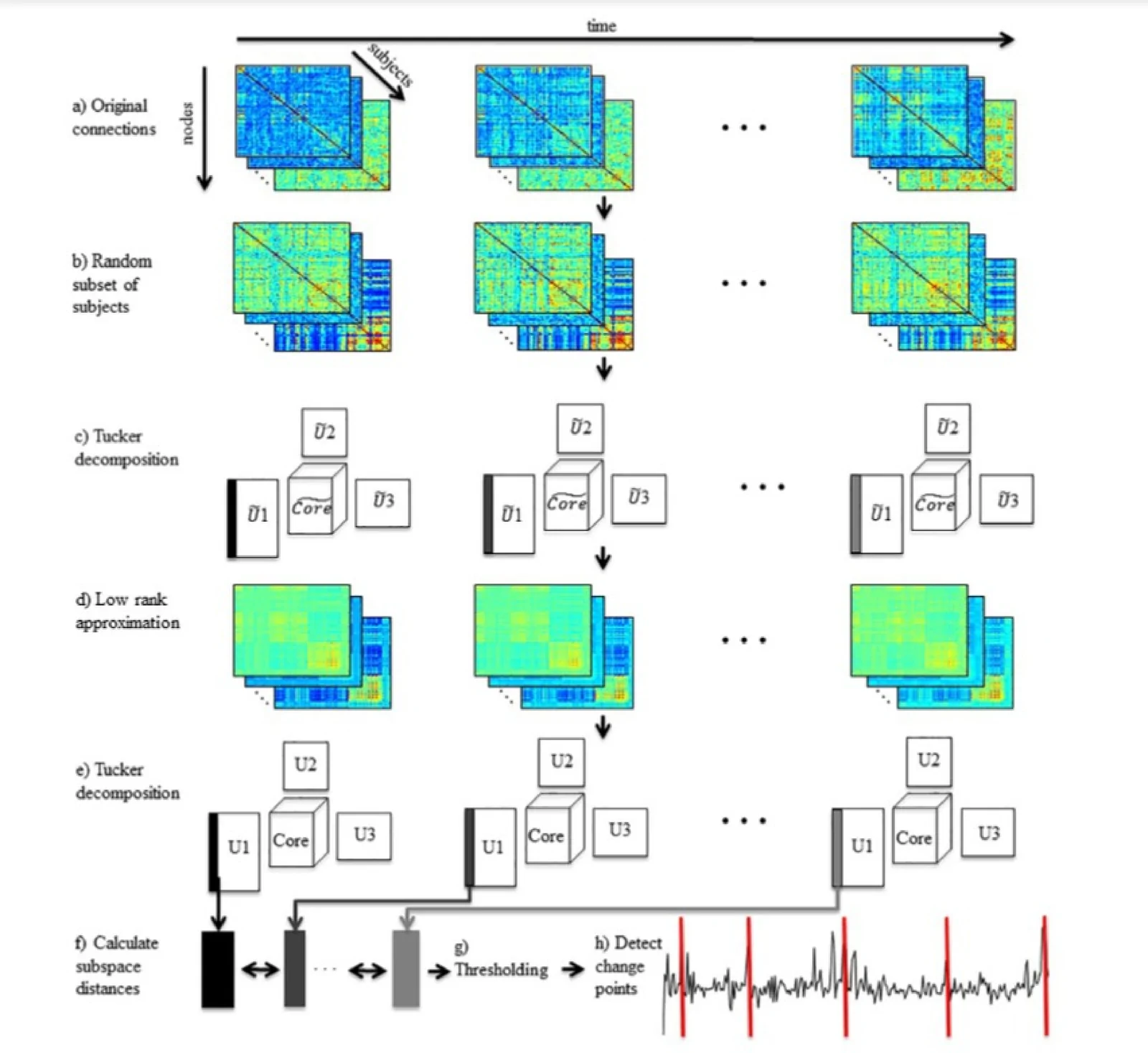
Flowchart of the model development, including elements such as the input layer and hidden layers.

We applied five iterations of random reshuffling along the patient axis to reduce ordering bias before decomposition. Then we applied Tucker decomposition to simplify the tensor and highlight the most important parameter interactions for each patient and the overall group. As illustrated in Figure 1, this process was carried out independently for data from Sirius and Pentacam devices to assess model performance across imaging systems.

### Evaluation Metrics

Discrimination performance was evaluated using standard classification metrics, which provide a comprehensive assessment of the model's ability to distinguish between eyes with KCN and healthy eyes. Sensitivity (true positive rate) was calculated as the proportion of actual positive cases correctly identified by the model and was computed using the following formula: Sensitivity = TP / (TP + FN), where TP denotes true positives, and FN denotes false negatives. Specificity (true negative rate) measured the proportion of actual negative cases accurately identified by the model and was calculated as: Specificity = TN / (TN + FP), where TN represents true negatives, and FP represents false positives.

Additionally, the area under the receiver operating characteristic curve (AUROC) was computed to quantify the model's overall discriminative capability across a range of decision thresholds. The ROC curve was generated by plotting sensitivity against the false positive rate (1 – specificity). AUROC values range from 0.5, indicating no discriminative ability, to 1.0, representing perfect discrimination. AUROC scores were calculated using established functions such as roc _auc _score from the scikit-learn library.

##  RESULTS

The proposed model demonstrated a strong capability to differentiate between patients with KCN and healthy individuals. Interestingly, our model consistently exhibited comparable levels of sensitivity and specificity, regardless of whether Pentacam or Sirius imaging was used. These metrics were evaluated separately for each subgroup, and the results are presented in Table 1. The sensitivity and specificity of our model when using Pentacam images were 88% and 90%, respectively. When using results from the Sirius device, the sensitivity and specificity were 92% and 96%, respectively.

**Table 1:** Diagnostic performance of the chaos-based AI model on Pentacam and Sirius tomography

**Subgroup**	**Sensitivity**	**Specificity**	***P*** **-value**
Pentacam	88%	90%	0.028
Sirius	92%	96%	0.025

### Comparison with Established Tomography Indices

To contextualize the performance of the chaos-based AI model, we compared its classifications with device-based indices derived from both Pentacam and Sirius tomography. All patients diagnosed with KCN by the Sirius “keratoconus” category were also correctly detected by the model. Among eyes labeled as “suspect” by Sirius, the model reclassified five eyes as KCN. Each of these eyes demonstrated abnormal Pentacam Belin–Ambrósio (BAD-D) values > 2.6 and met the ABCD criteria for KCN, confirming the validity of the model's classification. Agreement with the Pentacam BAD-D enhanced ectasia display was strong, with an AUC of 0.94 for differentiating KCN from healthy eyes. Similarly, the model showed close concordance with the PRFI, achieving an AUC of 0.92.^[21]^ These findings demonstrate that the proposed AI model not only aligns with established device-specific indices but also adds discriminatory power in borderline or suspect cases.

##  DISCUSSION

ML has emerged as an optimal computational technique for developing statistical models from extensive datasets. Most existing methods for diagnosing KCN rely on the human visual evaluation of colored maps and subjective assessments, leading to potential inconsistencies in diagnosis and treatment. By contrast, ML approaches can objectively analyze corneal imaging data, thereby assisting ophthalmologists in detecting early stages of KCN, identifying patients at high risk for future development of KCN, and planning treatment strategies.

The literature from 1994 to the present has documented the application of ML and deep learning models in detecting KCN. In this study, we introduced a new AI approach for detecting this condition using Pentacam and Sirius images. Our findings indicate that the proposed chaos-based AI model can automatically and accurately differentiate between patients with KCN and healthy individuals. This method achieved sensitivity between 88% and 92% and specificity between 90% and 96%.

According to Table 1, the model developed in this study outperforms traditional models that use convolutional neural network (CNN) deep learning methods. CNNs are widely used in ophthalmology and have become one of the most popular approaches for diagnosing and predicting the outcomes of KCN. Therefore, it was appropriate to compare our results with previous CNN-based work. As shown in Table 2, the proposed chaos-inspired AI model demonstrated diagnostic performance comparable to or exceeding that reported in several previously published ML approaches.

**Table 2:** Comparison of AI models, including the proposed chaos-based AI model, for KCN detection

**Author**	**Year**	**Device**	**Method**	**Sensitivity Mean–Max**	**Specificity Mean–Max**
Kato et al^[22]^	2021	Pentacam	Linear classification based on deep learning	77-88	60-70
Herber et al^[23]^	2021	Corvis ST	Classification based on machine learning	62-82	81-97
Chen et al^[24]^	2021	Pentacam	Amsler Krumeich classification	98-99	65-90
Present study	–	Pentacam, Sirius	Chaos-based model	88-92	90-96

Kato et al^[32]^ employed a linear method for disease estimation with sensitivity values ranging from 77.8% to 87.8% and specificity from 59.8% to 69.6%. Herber et al^[33]^ demonstrated that a nonlinear random forest classifier achieved higher predictive accuracy compared with linear discriminant analysis, supporting the idea that nonlinear methods better capture natural system variability. Chen et al^[34]^ reported very high sensitivity but relatively low specificity, raising concerns about possible overfitting. In contrast, the chaos-based model developed in the present study showed balanced and superior sensitivity and specificity. These findings highlight the promise of models inspired by chaos dynamics to better represent the complex, nonlinear properties of KCN.

Theoretical frameworks further reinforce this approach. The Kuramoto model of coupled oscillators and Strogatz's analyses of complex synchronization have been widely applied to biological systems, illustrating how small perturbations in initial conditions can generate large-scale nonlinear effects.^[35,36,7]^ These concepts align with the biological plausibility of applying chaos-inspired AI to corneal biomechanics and support the rationale for our modeling framework.

The present study also demonstrates that the chaos-based AI model can achieve performance comparable to, and in some cases exceeding, established device-based indices for KCN detection. In particular, the model showed excellent agreement with BAD-D and PRFI, both of which have been widely validated for ectasia screening.^[21]^ Furthermore, the model successfully reclassified a subset of eyes labeled as “suspect” by Sirius tomography into the KCN category. These cases consistently showed abnormal BAD-D values and ABCD criteria confirming the diagnosis. This highlights the potential of chaos-inspired AI not only to replicate device-specific algorithms but also to enhance diagnostic certainty in borderline cases. Previous comparative studies of Pentacam and Sirius tomography, such as that by Finis et al,^[11]^ have underscored variability between platforms. Our results suggest that the chaos-based AI framework may help bridge this gap by integrating multidimensional features in a manner aligned with clinical gold standards.

Recent meta-analyses further confirm the potential of AI models, particularly neural networks and Naïve Bayes classifiers, for KCN detection. These investigations consistently report sensitivity and specificity values above 0.90, which underscores the clinical promise of AI-based approaches across different imaging systems.^[38]^ More recent work by Ismael et al^[39]^ demonstrated that transformer-based models integrating multimodal data—including corneal topography, pachymetry, aberrometry, and biomechanical metrics—can significantly improve early KCN detection by capturing complex interdependencies often missed by conventional or unimodal AI frameworks. In a systematic review, Niazi et al^[40]^ noted that although most AI models exhibit strong diagnostic accuracy for clinical KCN, they often fall short in reliably detecting subclinical or suspect cases. This limitation emphasizes the potential advantage of chaos-based models, which are designed to capture nonlinear dynamics inherent to early KCN progression.

One important limitation of this study is the relatively small sample size, consisting of 113 participants, including 48 patients with KCN. Although the results are promising, the dataset may not fully represent the broader clinical population. Therefore, caution is warranted when generalizing these findings. Future multicenter studies with larger and more diverse cohorts are needed to further validate the robustness and generalizability of the proposed model across different imaging platforms and clinical settings.

In summary, given the nonlinear and complex nature of KCN progression and the interplay of biomechanical, genetic, and environmental factors, we developed a novel chaos-inspired AI-based system (Iran Model) capable of accurately detecting KCN. Although the preliminary results are promising, further validation in larger and multicenter cohorts is necessary.

##  Financial Support and Sponsorship

None.

##  Conflicts of Interest

None.
